# The Drunkard’s Daughter

**Published:** 1907-07

**Authors:** 


### The Drunkard's Daughter.

(By a young lady who was told that she was a monomaniac in her hatred of alcoholic liquors.)

Go, feel what I have felt,
  Go, bear what I have borne;
Sink 'neath a blow a father dealt,
  And the cold, proud world's scorn.
Thus struggle on from year to year,
Thy sole relief a scalding tear.

Go, weep as I have wept,
  O'er a loved father's fall;
See every cherished promise swept,
  Youth's sweetness turned to gall;
Hope fades, flowers strewn all the way
That led me up to the woman's day.

Go, kneel as I have knelt,
  Implore, beseech, and pray,
Strive the besotted heart to melt,
  The downward course to stay;
Be cast with bitter curse aside,
Thy prayers burlesqued, thy tears defied.

Go, stand where I have stood,
  And see the strong man bow;
With gnashing teeth, lips bathed in blood,
  And cold and livid brow;
Go, catch his wandering glance, and see,
There mirrowed his soul's misery

Go, hear what I have heard,
  The sobs of said despair,
As memory's feeling fount hath stirred,
  And its revealings there
Have told him what he might have been
Had he the drunkard's fate foreseen.

Go, to my mother's side,
  And her crushed spirit cheer;
Thine own deep anguish hide,
  Wipe from her cheek the tear;
Mark her dimmed eye, her furrowed brow,
The gray that streaks her dark hair now,

The toil-worn frame, the trembling limb,
And trace the ruin back to him,
Whose plighted faith, in early youth,
Promised eternal love and truth,
But who, foresworn, hath yielded up
This promise to the deadly cup,

And led her down from love and light,
From all that made her pathway bright,
And chained her there mid want and strife,
The lowly thing—a drunkard's wife;
And stamped on childhood's brow, so mild,
That withering blight—a drunkard's child;

Go, hear and see, and feel, and know
  All that my soul hath felt and known,
Then look within the wine-cup's glow;
  See if its brightness can atone;
Think of its flavor you would try,
If all proclaimed, Tis drink and die!

Tell me I hate the bowl!
  Hate is a feeble word;
I loathe, abhor, my very soul
  By strong disgust is stirred
Whene'er I see, or hear, or tell
Of the DARK BEVERAGE OF HELL!

